# EBV and multiple sclerosis: expression of LMP2A in MS patients

**DOI:** 10.3389/fnins.2024.1385233

**Published:** 2024-04-24

**Authors:** Simone Agostini, Roberta Mancuso, Domenico Caputo, Marco Rovaris, Mario Clerici

**Affiliations:** ^1^IRCCS Fondazione Don Carlo Gnocchi ONLUS, Milan, Italy; ^2^Department of Pathophysiology and Transplantation, University of Milan, Milan, Italy

**Keywords:** multiple sclerosis, Epstein–Barr virus, EBNA-1, LMP2A, antibodies, mRNA, rehabilitation

## Abstract

Several evidences, including increased serum titers of Epstein–Barr virus (EBV)-specific antibodies and the presence of EBV DNA in brain of patients suggest a possible role of this virus in the pathogenesis of Multiple Sclerosis (MS), a chronic neurodegenerative disease with an unknown etiopathology. Aim of the present study is to verify if the expression of LMP2A and EBNA-1, two EBV genes, is altered in MS patients. EBV viral load, LMP2A and EBNA-1 gene expression and EBNA-1 antibodies titers were evaluated in blood of EBV-seropositive MS patients (*n* = 57; 31 relapsing remitting –RRMS- and 26 progressive -PMS-patients) and age- and sex-matched healthy controls (HC, *n* = 49). Results showed that EBNA-1 and VCA antibodies titers are significantly augmented in MS patients compared to HC (*p* < 0.05 for both antibodies); detection of EBV DNA was more frequent as well in MS patients compared to HC, although without reaching statistical significance. Regarding viral gene expression, LMP2A was significantly more frequently detected and more expressed in MS patients compared to HC (*p* < 0.005) whereas no differences were observed for EBNA-1. Considering patients alone, EBNA-1 was significantly more frequent in PMS compared to RRMS (*p* < 0.05), whereas no differences were observed for LMP2A. Increased expression of the LMP2A latency-associated gene in MS patients supports the hypothesis that EBV plays a role in disease etiopathology.

## 1 Introduction

Multiple Sclerosis (MS) is a chronic inflammatory disease of the central nervous system (CNS) which is the most common cause of neurological disability in young adults, affecting over 2.5 million people worldwide (Dobson and Giovannoni, 2019). It is known that an interplay of genetic and environmental factors leads to the chronic activation of immune cells resulting in neuronal injury, and several epidemiological studies identified a number of possible environmental risk factors in MS, including infection with viruses such as Epstein Barr virus (EBV), herpes simplex virus (HSV) type 1 and 2, human herpesviruses 6 (HHV-6), measles, mumps and rubella (Tarlinton et al., 2020).

EBV (also called human herpesvirus 4, HHV-4) is a ubiquitous human virus with a double stranded DNA genome (about 175 kb long) wrapped in a protein capsid and belonging to the *Herpesviridae* family, *Gammaherpesvirinae* subfamily, *Lymphocryptovirus* genus (Baer et al., 1984; Young and Rickinson, 2004). The virus is spread worldwide and is harbored persistently by almost all adults, regardless of health status. In western countries with high standard of living primary EBV infection occurs commonly in adolescence and can manifest as mononucleosis (IM) in 30–50% of cases (Junker, 2005). After primary infection, a phase during which the virus replicates in oro-pharyngeal epithelial cells, EBV establishes a persistent and lifelong latent infection in circulating B lymphocytes.

The possible real role of EBV in MS is not completely clear, as at now several discordant and often opposite results are found and published (Lassmann et al., 2011; Soldan and Lieberman, 2023). In particular, the presence of EBV in MS brain tissue or CSF could (Hassani et al., 2018; Moreno et al., 2018) or could not (Willis et al., 2009; Peferoen et al., 2010; Sargsyan et al., 2010) be observed. Results of a recent longitudinal study on a very large population (10 million of subjects), though, showed that EBV infection precedes the increase of serum neurofilament light chain (NfL) (an indicator of neuroaxonal degeneration) and is a trigger for the development of MS (Bjornevik et al., 2022).

During latency, EBV expresses genes in four different programs, called latency 0, I, II and III (Munz, 2019; Murata et al., 2021). In latent phase III all the EBV latent genes are expressed, the infected B cell is activated and enters the germinal center. The next step is the shift of EBV into latent phase II, in which only the LMP1, LMP2A, LMP2B and EBNA-1 proteins are expressed. The expression of these proteins drives EBV-infected memory B cell into the latency I phase in which only EBERs genes are expressed. The latency I phase is observed only during the homeostatic proliferation of memory cells; in this phase EBERs as well EBNA-1 genes are expressed.

LMP2A is a 5,856 base pairs (bp) gene that generates a mRNA formed by 9 exons. The correspondent protein (52.9 KDa) is 497 aminoacids (aa) long and includes 12 hydrophobic membrane-spanning domains, a 27 aa cytosolic carboxyl-terminus involved in interclustering, and another 119 aa cytosolic sequence endowed with signaling capabilities (Young and Rickinson, 2004; Pang et al., 2009). LMP2A is one of the few EBV proteins required for establishing and maintaining viral latency (Miller et al., 1995), and has the potential to transform epithelial cells, to modulate cell signal transduction (Liang et al., 2017), to inhibit cell proliferation and migration (Jiang et al., 2021), to inhibit NF-kB pathway (Stewart et al., 2004), and to affect the CD8+ T cell recognition against EBV-infected cells (Rancan et al., 2015).

The EBNA-1 gene is 1922 bp long and translates a 641 aa homodimeric protein (56.4 KDa) which binds site-specifically to a 16 bp DNA sequence (Baer et al., 1984; Frappier and O’Donnell, 1991), and maintains EBV as episome in the nucleus. By binding EBNA-1-binding sites within the EBV latent origin of replication (OriP), EBNA-1 mediates genome synthesis and acts as a viral transcriptional transactivator of latent genes.

EBV could play a role in MS pathogenesis of Multiple Sclerosis in two possible ways: (1) molecular mimicry as a consequence of cross-reactions between EBNA-1 antigens and human myelin antigens (Lanz et al., 2022); (2) CD8+ T lymphocyte-mediated immune response, as suggested by increased EBV-specific CD8+ T-lymphocytes and EBNA-1-specific serum antibodies titers in MS patients (Jilek et al., 2008; Angelini et al., 2013). The expression pattern of EBV latent genes is not yet well investigated in MS; aim of the present study is to analyze LMP2A and EBNA-1 expression of latent genes in MS patients in the attempt to shed more light on the possible association between disease and EBV infection.

## 2 Materials and methods

### 2.1 Study population and specimens

A total of 106 individuals were enrolled in the study: 57 were patients with a diagnosis of MS according to the 2010 revised McDonald criteria (Polman et al., 2011). All the patients were followed by the Multiple Sclerosis Unit of the IRCCS Fondazione Don Gnocchi, Milan, and were undergoing different rehabilitative programs; the other 49 individuals were age- and sex-matched healthy controls (HC). Diagnosis was confirmed by magnetic resonance imaging (MRI). Thirty-one of the 57 patients had a diagnosis of relapsing–remitting disease (RRMS), whereas 26/57 were patients with progressive form (PMS) (Thompson et al., 2018; Jakimovski et al., 2024). Regarding the PMS, the majority of the patients had a diagnosis of secondary progressive (SPMS, 20/26), whereas the remaining ones had a diagnosis of primary progressive MS (PPMS, 6/26) (Thompson et al., 2018).

The study complies with the ethical principles of the Declaration of Helsinki; written informed consent was provided by all the individuals involved in the study and was prepared according to a protocol approved by the local ethics committee of the IRCCS Fondazione Don Carlo Gnocchi.

### 2.2 Blood sample collection

Whole blood was collected by venipuncture. Serum was obtained by centrifugation for 10 min at 2.000g at room temperature and aliquoted into sterile cryovials stored at −20°C. Serum was utilized to measure antibody titers; whole blood was utilized for DNA and RNA extraction.

### 2.3 EBV antibodies detection

EBV seropositivity was tested measuring IgG anti-EBV nuclear antigen 1 (EBNA-1) as well EBV viral capsid antigen (VCA) by commercial enzyme immunoassay kits (EBV EBNA-1 IgG IBL, Tecan, Hamburg, Germany, and EBV VCA IgG IBL, Tecan), according to the manufacturer instructions. The optical density (OD) was determined at 450 nm by plate reader (Sunrise, Tecan, Mannedorf, Switzerland).

### 2.4 DNA extraction and EBV viral load

DNA was extracted from whole blood using a standard phenol/chloroform procedure; 50 ng were used to measure EBV viral load (the amplificated region is EBNA-LP) by qPCR (CXF96 Bio-Rad, Hercules, CA, US) as previously reported (Agostini et al., 2018).

### 2.5 RNA extraction and mRNA analysis

Total RNA was extracted from fresh peripheral blood using the QIAMP RNA Blood Mini kit (Qiagen, Hilden, Germany) within 1 h from blood collection and following manufacturer’s instruction.

mRNA concentration was determined measuring the optical density (OD) at 260 nm with a spectrophotometer. Purity was determined as the 260 nm/280 nm, as well as 260 nm/230 nm OD ratio with expected values between 1.8 and 2.0 indicating absence of protein contamination. Extracted RNA was immediately treated with TURBO DNA-free DNase (Ambion INC, Austin, TX, United States).

Because EBNA-1 and LMP2A viral genes expression in whole blood is limited (Serafini et al., 2010) a two-step selected preamplification qPCR was performed. In the first step EBNA-1 and LMP2A mRNA was retrotrascribed in cDNA using the iScript One-Step RT-PCR kit (Bio Rad, Hercules, CA, US), according to the manufacturers’ instructions. In the second step, 5 μL of cDNAs underwent TaqMan-qPCR assay. Primers and probes are reported in Supplementary Table S1. mRNA relative quantification is reported as deltaCt, calculated as the difference between the Ct obtained from a positive control (plasmid containing the LMP2A DNA, obtained from EBV^+^ B95.8 cell lines) and the Ct of the analyzed sample.

### 2.6 Statistical analysis

Normally distributed data were summarized as mean and standard deviation (SD), and comparison among groups were analyzed by ANOVA test and Student t-test. Not-normally distributed data were summarized as median and Interquartile Range (IQR: 25^th^ and 75^th^ percentile), and comparisons were analyzed by Kruskal-Wallis test and Mann Whitney U test, as appropriate. Correlations were analyzed by Spearman’s correlation coefficient. Qualitative data were compared using Chi-squared test.

## 3 Results

### 3.1 Clinical characteristics of study subjects

Clinical characteristics of all the individuals enrolled in the study are summarized in Tables 1, 2. Age and sex distribution were comparable between MS patients and HC. As expected, the EDSS score was significantly higher in PMS (6.30 ± 1.10) than in RRMS (3.17 ± 1.81; *p* < 0.0001) patients. Disease onset was significant earlier in RRMS (23.13 ± 10.98 years) compared to PMS (36.92 ± 11.29 years; p < 0.0001), but disease duration was similar in both groups of MS patients (15.81 ± 10.15 years for RRMS; 18.54 ± 11.05 years for PMS). Most patients (43/57) were drug-free for at least 2 months preceding enrollment; 12 were treated with immunomodulatory compounds (i.e., natalizumab and glatiramer acetate) whereas 1 individual received immunosuppressive treatment (azathioprine).

**Table 1:** Demographic and clinical characteristics of subjects enrolled for the study.

	Multiple Sclerosis patients	Healthy controls
N	57	49
Gender (M:F)	22:35	20:29
Age, years	45.98 ± 12.64	47.14 ± 8.37
Age at onset, years	29.54 ± 13.03	---
Disease duration, years	17.05 ± 10.56	---
EDSS	4.60 ± 2.21	---

Data are expressed as means ± standard deviation; N, number; M, male; F, female; EDSS, Expanded Disability Status Scale.

**Table 2:** Demographic and clinical characteristics of Multiple Sclerosis patients.

	RRMS patients	PMS patients
N	38	29
Gender (M:F)	16:22	12:17
Age, years	39.8 ± 8.1	54.0 ± 11.1
Age at onset, years	20.0 ± 10.4	37.2 ± 9.3
Disease duration, years	19.9 ± 12.8	17.6 ± 9.9
EDSS	3.3 ± 2.1	6.4 ± 1.4

Data are expressed as means ± standard deviation; RRMS, relapsing remitting multiple sclerosis patients; PMS, progressive multiple sclerosis patients; N, number; M, male; F, female; EDSS, Expanded Disability Status Scale.

### 3.2 EBV seropositivity

All the 106 subjects included in the study were IgG EBNA-1- and VCA- seropositive. EBNA-1- and VCA- specific antibodies titers were significantly higher in MS patients (EBNA-1: 965.00; 176.00–53336.57 U/mL; VCA: 376.10; 24.56–2063.72 U/mL) compared to HC (EBNA-1: 962.44; 895.58–993.70 U/mL; VCA: 296.93; 37.43–1051.32 U/mL) (*p* = 0.03 for both comparisons), confirming data previously reported (Agostini et al., 2018; Dominguez-Mozo et al., 2022). No differences were observed for EBNA-1 and VCA antibodies concentration between PMS and RRMS, and also when PMS patients were divided into those with either a diagnosis of SPMS or PPMS; no correlations were observed between these parameters and EDSS, age at onset and disease duration.

### 3.3 EBV viral load

EBV DNA was more frequently detected in MS patients (33%) compared to HC (28%), but the viral load was similar in the two groups (59.91 copies/μg vs. 58.44 copies/μg). No associations were found between EBV viral load and antibodies titers, as well as with EDSS, age at onset and disease duration.

### 3.4 LMP2A and EBNA-1 gene expression

**Figure 1:**
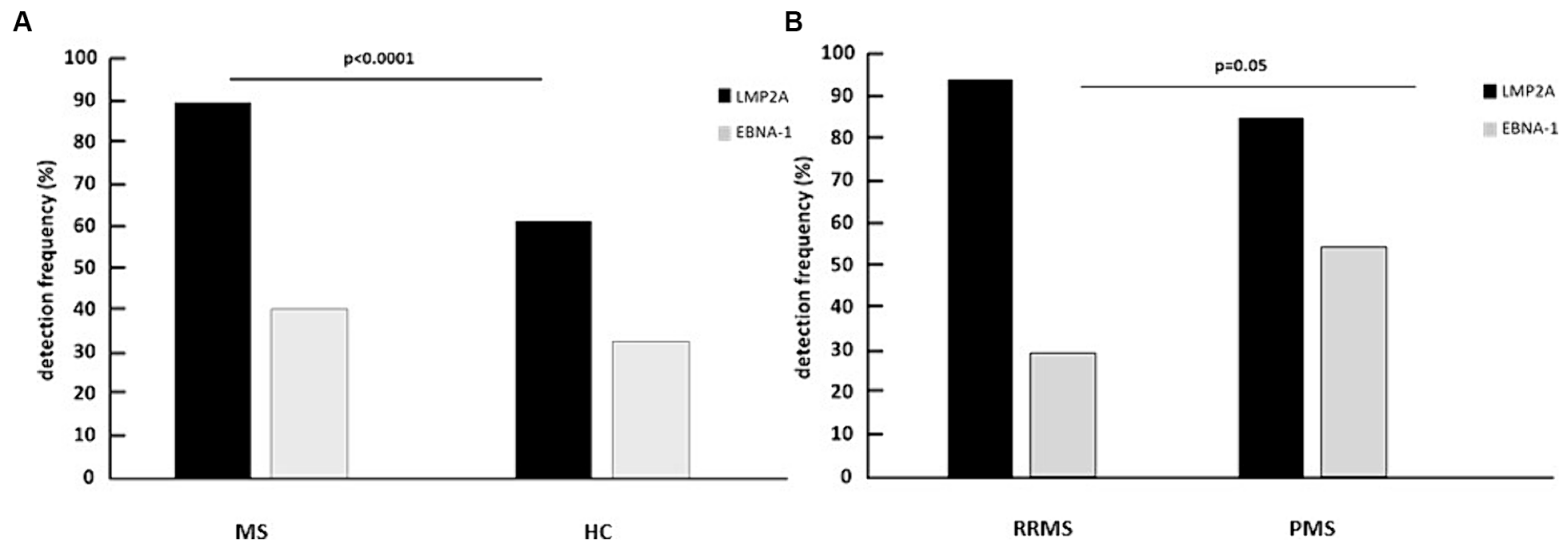
Percentage of multiple sclerosis patients (MS) and healthy controls (HC) **(A)** and of relapsing remitting multiple sclerosis patients (RRMS) and progressive multiple sclerosis patients (PMS) **(B)** expressing LMP2A and EBNA-1 mRNA in whole blood.

LMP2A was significantly more frequently observed in MS patients (89%) compared to HC (61%, *p* < 0.0001); EBNA-1 was more frequently seen as well in MS patients compared to HC (40% vs. 32%), even if differences did not reach statistical significance (Figure 1A). Importantly, the frequency of LMP2A and EBNA-1 mRNA co-expression was significantly higher in MS patients compared to HC (*p* = 0.004; Supplementary Table S2). LMP2A was more frequently observed in PMS (94%; SPMS: 95%; PPMS: 93%) compared to RRMS (85%); differences with HC persisted even when the two clinical phenotypes were separately compared with HC (PMS vs. HC: *p* = 0.03; RRMS vs. HC: *p* = 0.001). Notably, EBNA-1 was significantly more frequently detected in PMS (54%) than in RRMS (29%) (*p* < 0.05) (Figure 1B). Dividing PMS patients into those with either a diagnosis of SPMS or PPMS, EBNA-1 frequency detection remained higher in both groups compared to RRMS patients (SPMS: 50%; *p* < 0.05; PPMS: 67%). No differences were observed considering the simultaneous detection of the two mRNAs in PMS and RRMS, or considering PPMS and SPMS separately.

**Figure 2:**
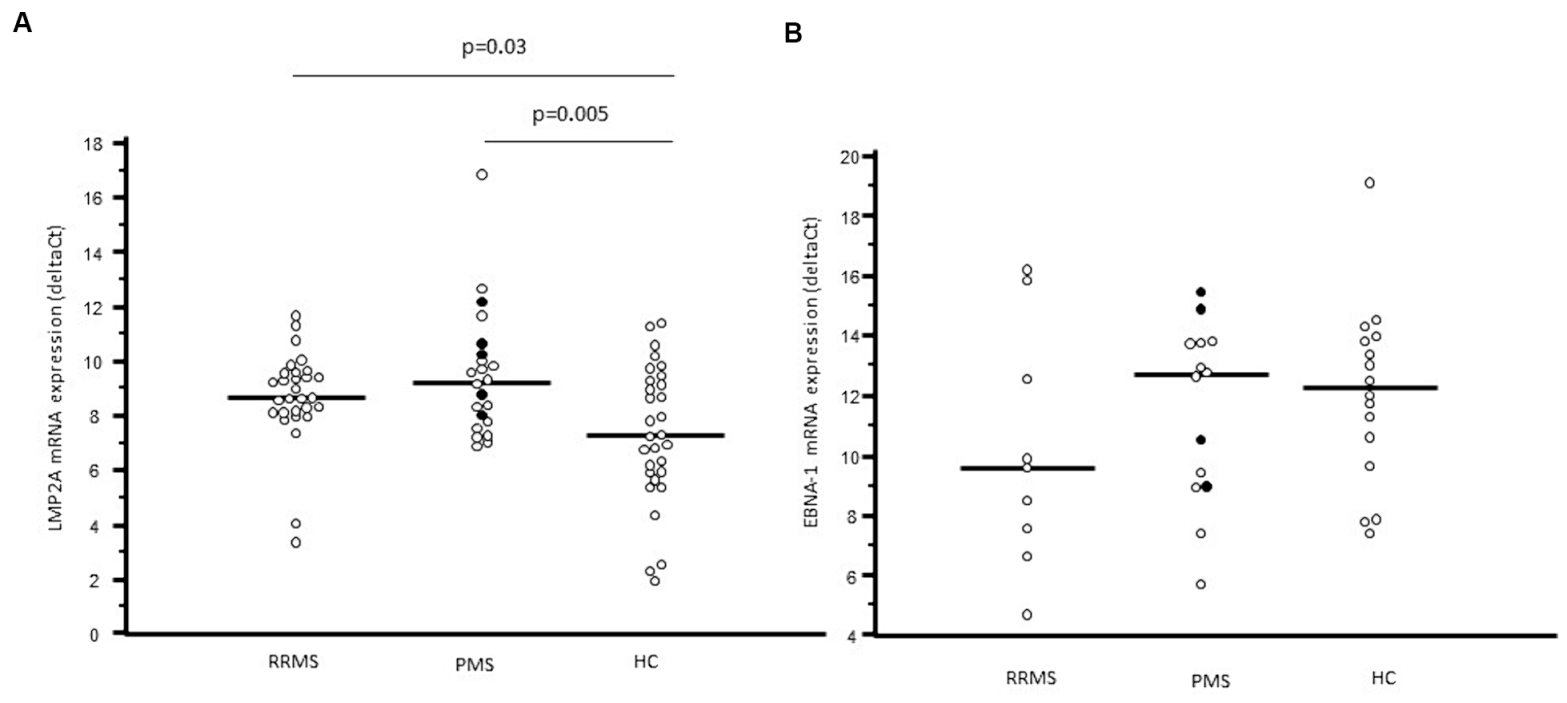
LMP2A mRNA expression **(A)** and EBNA-1 mRNA expression **(B)** in whole blood of relapsing remitting multiple sclerosis patients (RRMS), progressive multiple sclerosis patients (PMS) and healthy controls (HC). In PMS columns, black plots represent primary progressive multiple sclerosis patients (PPMS), whereas white dots represent secondary progressive multiple sclerosis patients (SPMS).

LMP2A gene expression was significantly increased as well in blood of MS patients (9.00; 8.05–9.79 deltaCt) compared to HC (mRNA: 7.29; 5.90–9.26 deltaCt, p = 0.004) whereas EBNA-1 gene expression was similar in the two groups (mRNA: 10.55; 8.58–13.74 vs. 12.26; 10.10–13.90 deltaCt) (Figure 2). Interestingly, in MS patients, but not in HC, a significant positive correlation was observed between LMP2A and EBNA-1 gene expression (*p* = 0.01) (Supplementary Figure S1).

LMP2A gene expression was similar in RRMS and PMS (mRNA: 8.65, 8.10–9.57 vs. 9.22; 7.78–10.24 deltaCt) and was significantly increased compared to the values observed in HC (RRMS vs. HC: *p* = 0.003; PMS vs. HC: *p* = 0.0005). The statistical difference remains also splitting PMS in SPMS (*p* = 0.02 vs. HC) and in PPMS (*p* = 0.03 vs. HC) (Figure 2A). In contrast with these results, EBNA-1 gene expression was comparable in PMS, RR-MS and HC (Figure 2B). No correlation was found between EBNA-1 and LMP2A gene expression in RRMS or PPMS, this is probably due to the overall small number of patients in each group.

None of these comparisons changed if treated patients were excluded from analyses, whereas the statistical differences disappeared when MS patients undergoing therapy alone were analyzed; this was probably due to the overall small number of patients that we could analyze.

Finally, no correlations could be detected between EDSS, age at onset, disease duration and EBNA-1 and LMPA2 expression or antibody concentration.

## 4 Discussion

The presence of a possible correlation between EBV infection and MS is controversial and studies published in recent years could not definitely clarify whether this virus indeed plays a role in the disease. Increased severity of symptoms in transgenic experimental autoimmune encephalomyelitis (EAE) mice, the animal model of human CNS demyelinating diseases including MS, is associated with the presence of LMP2A EBV gene-expressing B lymphocytes (Chang et al., 2012). Starting from this observation, we decided to analyze LMP2A and EBNA-1 expression in peripheral lymphocytes of MS patients. Results herein are, to the best of our knowledge, the first to report that LMP2A mRNA is more frequently detected and is upregulated in MS patients compared to HC, whereas no important differences could be seen when EBNA-1 was analyzed.

LMP2A is a 12-transmembrane domain protein encoded by EBV that significantly modifies the activation and survival of host B lymphocytes (Portis and Longnecker, 2000; Swanson-Mungerson and Longnecker, 2007). LMP2 mRNA was found in EBV-infected circulating B lymphocytes of both MS and healthy individuals (Tierney et al., 1994; Serafini et al., 2007), but, importantly, LMP2A mRNA was also found in brain-infiltrating B lymphocytes of MS patients (Serafini et al., 2010). Notably, LMP2A proteins were recently observed to be carried inside serum-derived exosomes of both MS patients and HC, but, concentrations were significantly increased in exosomes of patients (Mrad et al., 2021). Finally, CNS-infiltrating CD8+ T lymphocytes of MS patients were shown to recognize the majority of EBV latent proteins, LMP2A included (Serafini et al., 2019).

B lymphocytes are known to play a pivotal role in MS (reviewed by Comi et al., 2021), but the mechanisms involved in MS-associated pathogenic processes are still only partially clarified. Because EBV infection establishes latent infection in memory B lymphocytes, the presence of LMP2A inside such lymphocytes could have a key role in MS. Thus, increased LMP2A expression could stimulate the production of antibody by autoreactive B cells upon mimicking a BCR signal (Wang et al., 2006), deregulating T cell responses and damaging CNS tissues. Moreover, LMP2A could also enhance antigen presentation by autoreactive B cells that have bound antigens, causing the activation of autoreactive T cells. Notably, besides modulating the activity of B lymphocytes, LMP2A could have an additional role in the pathogenesis of MS. Thus, EBV infection causes ERK/MAPK over-activity (Roberts and Cooper, 1998), ultimately resulting in the excessive production of pro-inflammatory cytokines (Ten Bosch et al., 2021). Two distinct sets of data showed that, during EBV latent infection, LMP2A can constitutively activate the ERK/MAPK pathway (Anderson and Longnecker, 2008; Pan et al., 2008) and, in this way, contribute to MS-associated inflammation.

Interestingly, in our cohort of MS patients a positive correlation was found between LMP2A and EBNA-1 gene expression. This association is in apparent contrast with the findings obtained by Rancan and coworkers, that, in an *in vitro* study, showed a negative relation between LMP2A and EBNA1 mRNA expression (Rancan et al., 2015), but in line with that observed by Mrad and coworkers, that found a strong positive correlation between LMP2A and EBNA-1 protein concentration in serum-derived exosome (Mrad et al., 2021). These conflicting results can be due to the host genotype pattern, in particular regarding HLA alleles, but other studies are needing to better clarify this aspect.

Regarding antibodies, serum concentration of the EBV-specific antibodies VCA and EBNA-1 was significantly increased as well in MS patients, confirming data already present in literature (Agostini et al., 2018; Dominguez-Mozo et al., 2022). Notably, we observed that although EBNA-1 antibody serum detection frequency was comparable between MS patients and HC, a significant higher frequency was seen in PMS compared to RRMS. Although this observation needs to be confirmed in ampler cohorts, it could indicate that EBNA-1 antibody serum detection is a marker of severity and disease progression, as had been previously suggested (Lunemann et al., 2010; Kvistad et al., 2014). To note, we detected EBNA-1 mRNA in less than half of the enrolled population: this could be due to the very low percentage of EBV-infected peripheral blood mononuclear cells (Cocuzza et al., 2014). This finding needs to be further analyzed, also considering that the percentage of individuals in whom LMP2A was detected, was much higher.

These results are preliminary and present some weaknesses – mainly the small number of SM patients examined – nonetheless we believe that these results could be of interest to the research community as they shed some light on the complexity of the relationship between MS and EBV. Questions that could stem from our results and need to be clarified are: (1) a better characterization of host HLA genetic pattern; (2) analysis of vitamin D concentration, a molecule that seems to be strictly associated with EBV-specific infection in MS (Agostini et al., 2018; Guan et al., 2019); (3) analysis of other latency EBV genes; (4) analysis of EBV viral load, and of LMP2A and EBNA-1 mRNA expression in the B lymphocytes alone (i.e. the natural EBV cellular host); (5) analysis of ERK/MAPK pathway; and (6) analysis of the functional effects of LMP2A overexpression on activation of the different types of immune cells. In conclusion, we observed that the EBV LMP2A gene is overexpressed in MS patients regardless of the clinical phenotype, whereas the expression of EBNA-1 is increased in PMS alone. These data, although in need of confirmation in independent and bigger cohorts, offer support to the hypothesis that EBV infection might play a role in the pathogenesis of Multiple Sclerosis.

## Data availability statement

The raw data supporting the conclusions of this article will be made available by the authors, without undue reservation.

## Ethics statement

The studies involving humans were approved by the local ethics committee of the IRCCS Fondazione Don Carlo Gnocchi. The studies were conducted in accordance with the local legislation and institutional requirements. The participants provided their written informed consent to participate in this study.

## Author contributions

SA: Conceptualization, Data curation, Formal analysis, Investigation, Visualization, Writing – original draft, Writing – review & editing. RM: Conceptualization, Formal analysis, Writing – original draft, Writing – review & editing. DC: Investigation, Writing – review & editing. MR: Investigation, Writing – review & editing. MC: Investigation, Writing – original draft, Writing – review & editing.
