# Supplementary material for: EBV and multiple sclerosis: expression of LMP2A in MS patients
Source: Front Neurosci. 2024 Apr 24;18:1385233. doi: 10.3389/fnins.2024.1385233 (PMC11076709; doi:10.3389/fnins.2024.1385233)

Supplementary Material

Supplementary Figure 1. Correlation between LMP2A and EBNA-1 mRNA expression in whole blood of multiple sclerosis patients.

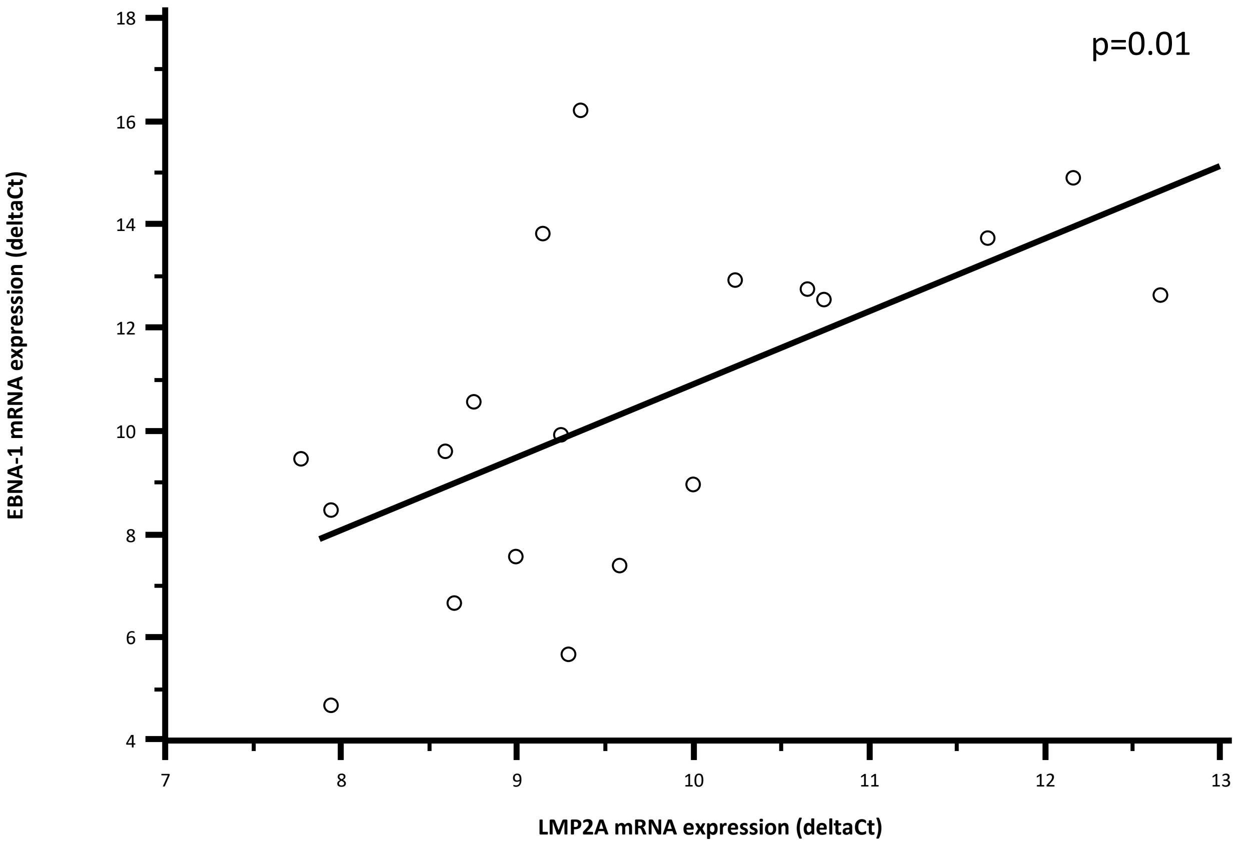

Supplement: Supplementary file 1 [file Image_1.pdf]
