# Supplementary material for: EBV and multiple sclerosis: expression of LMP2A in MS patients
Source: Front Neurosci. 2024 Apr 24;18:1385233. doi: 10.3389/fnins.2024.1385233 (PMC11076709; doi:10.3389/fnins.2024.1385233)
Supplement: Supplementary file 2 [file Table_1.DOCX]

Supplementary Material

# Supplementary Table 1. Primers and probes utilized for qPCR

| **Primers and probes** | **nucleotides** | **Sequence 5‘-3‘** |
| --- | --- | --- |
| LMP2AEXTF | 985-1005* | 5’-CTTCGTGCTCTTCATGTCCA-3’ |
| LMP2AEXTR | 1303-1283* | 5’-CTATTTCCACTGCCCCATTC-3’ |
| LMP2AINTF | 1011-1033* | 5’-AAGATCCTTCTGGCACGACTGT-3’ |
| LMP2AINTR | 1150-1124* | 5’–TGCAGAACAAATTGGGTATAAATTCA-3’ |
| LMP2A PROBE | 1043-1069* | FAM-TCTCGCACTCTTGTTGCTAGCCTCCG-TAMRA |
| EBNA1EXTF | 375-396^#^ | 5’-TTCCCTGGGAGAGAGCTGATT-3’ |
| EBNA1EXTR | 1036-1016^#^ | 5’-CTCCTCGTCCTCGTCCTCTT-3’ |
| EBNA1INTF | 428-448^#^ | 5’-GGAGCCTGACCTGTGATCGT-3’ |
| EBNA1INTR | 927-905^#^ | 5’TAGGCCATTTCCAGGTCCTGTA-3’ |
| EBNA PROBE | 490-514^#^ | FAM-CGCGGCCGTCTCCTTTAAGTGTGA-TAMRA |

*nt. position referred to accession number: GU979730.1

# nt. position referred to accession number: M12553.1
