# Supplementary material for: EBV and multiple sclerosis: expression of LMP2A in MS patients
Source: Front Neurosci. 2024 Apr 24;18:1385233. doi: 10.3389/fnins.2024.1385233 (PMC11076709; doi:10.3389/fnins.2024.1385233)
Supplement: Supplementary file 3 [file Table_2.DOCX]

Supplementary Material

# Supplementary Table 2. Detection of LMP2A and EBNA-1 mRNA in study population.

|  | **Multiple Sclerosis patients (57)** | **Healthy controls (49)** |
| --- | --- | --- |
| **LMP2A^+^ and EBNA-1^+^** | 19  (33%) | 9  (18%) |
| **LMP2A^+^ and EBNA-1^-^** | 32  (56%) | 21  (43%) |
| **LMP2A^-^ and EBNA-1^+^** | 4  (7%) | 7  (14%) |
| **LMP2A^-^ and EBNA-1^-^** | 2  (4%) | 12  (25%) |

Values are expressed as absolute number and percentage.
